# Emotional intelligence as a predictor of self-efficacy among students with different levels of academic achievement at Kermanshah University of Medical Sciences

**Published:** 2015-04

**Authors:** AMENEH GHARETEPEH, YAHYA SAFARI, TAHEREH PASHAEI, MANSOUR RAZAEI, MOHAMMAD BAGHER KAJBAF

**Affiliations:** 1 School of Health, Kermanshah University of Medical Sciences, Kermanshah, Iran;; 2 School of Paramedical Sciences, Kermanshah University of Medical Sciences, Kermanshah, Iran;; 3 School of Health, Kurdistan University of Medical Sciences, Sanandaj, Iran;; 4 Department of Educational Science and Psychology, Isfahan University, Isfahan, Iran;

**Keywords:** Emotional intelligences, Self efficacy, Achievement

## Abstract

**Introduction:**

studies have indicated that emotional intelligence is positively related to self-efficacy and can predict the academic achievement. The present study aimed to investigate the role of emotional intelligence in identifying self-efficacy among the students of Public Health School with different levels of academic achievement.

**Methods:**

This correlational study was conducted on all the students of Public Health School. 129 students were included in the study through census method. Data were collected using Emotional Intelligence and self-efficacy questionnaires and analyzed using descriptive statistics and regression analysis by SPSS 14.

**Results:**

The average score of students with high academic achievement was higher in self-efficacy (39.78±5.82) and emotional intelligence (117.07±10.33) variables and their components than that of students with low academic achievement (39.17±5.91, 112.07±13.23). The overall emotional intelligence score to predict self-efficacy explanation was different among students with different levels of academic achievement (p<0.001). Self-efficacy structure was explained through self-awareness and self-motivation components in students with low academic achievement (r=0.571). In students with high academic achievement, self-awareness, self-motivation and social consciousness played an effective role in explaining self-efficacy (r=0.677, p<0.001).

**Conclusion:**

Emotional intelligence and self-efficacy play an important role in achieving academic success and emotional intelligence can explain self-efficacy. Therefore, it is recommended to teach emotional intelligence skills to students with low academic achievement through training workshops.

## Introduction

Intelligence quotient (IQ) has long been considered as the main factor of academic success and achievement, but some critics assert that intellectual abilities are too much emphasized in IQ opinions (1). Findings of a research conducted by Life showed that general intelligence explains only 50 percent of academic achievement (2). Research has shown that factors other than cognitive intelligence or talent affect professional life and academic success (3). More intelligent individuals were significantly more stable in their education (4). Some variables, such as social intelligence, are known as important factor STO control emotions and others as factors to distinguish between them and use information to direct thinking and action (5).

Emotional intelligence is a concept first introduced by Meyer and Maludy in the early 1990s (6). This element is a set of linked cognitive and emotional abilities (7). This cognitive structure has four components: emotional self-assessment, self-expression assessment, identification of others’ emotions for emotional self-regulation, and the use of emotion to facilitate performance (8).The findings of a study indicated that emotional intelligence had twice the power of cognitive intelligence to predict academic achievement explanation (9). But in another study, EI did not appear to reliably predict future academic performance. Future studies should define the role of EI in admission decisions (10).

In a study conducted, a positive relationship was reported between emotional intelligence and academic motivation, and students with high, medium and low motivation had significantly different emotional intelligence (11). On the other hand, people with high emotional intelligence are those who are capable of adapting to situations of life and use effective coping skills while facing problems (12). Therefore, it can be said that they own the element of self-efficacy (3). Self-efficacy is one of the social cognitive factors discussed in Albert Bandura’s theory.  Bandura has defined self-efficacy as one’s belief in one’s ability to succeed at tasks (13).

Effective performance requires both skills and one’s belief in one’s abilities to do the tasks. Therefore, exchanges with the environment are somewhat affected by people’s judgments about their abilities (14). Several studies have approved the potential of self-efficacy to predict academic achievement (15, 16) Research has indicated that there is a positive relationship between the feeling of self-efficacy and responsibility for homework, high GPA at semester-end exams (17), teachers’ job satisfaction, and students’ academic achievement (18). A study conducted by Amini (15) showed that self-efficacy explained 21% of academic achievement.  Identification of factors affecting academic success is considered since students’ academic failure imposes enormous financial and spiritual expenses on the educational system, and academic failure seriously threatens the learners’ public and mental health while decreasing their motivation and self-confidence (19).

Emotional intelligence and self-efficacy are two important structures to be taken into account while studying the causes of academic success or failure. These structures are flexible and improvable through necessary interventions. On the other hand, research has indicated that emotional intelligence is positively related to self-efficacy and both of these variables can predict each other (20). Research findings have also shown that stress management failure and increased ineffective anxiety and stress are direct results of low self-efficacy (21). People not believing in their abilities get disappointed while facing risky circumstances and are less likely to operate effectively. Such people are afraid of dealing with challenging issues and consequently their performance is negatively affected, leading to more feeling of inadequacy (22).

On the other hand, severe anxiety can lead to decreased performance and consequently decreased feeling of self-efficacy. Therefore, a person with high emotional intelligence can necessarily control his emotions and deal with problems favorably (23). Accordingly, if we cannot consider self-efficacy as a component of emotional intelligence, we should not at least ignore its high overlap. Studies have emphasized that more research is needed to clarify which components of emotional intelligence play a more important role in explaining self-efficacy changes (3) and which ones have causal effects (23). Therefore, conducting the present study is important because, in addition to comparing the students’ (with high and low academic achievement) level of access to emotional intelligence and self-efficacy components, it clarifies whether emotional intelligence can explain self-efficacy, and if so, which components of emotional intelligence play a more important role in explaining self-efficacy in the above-mentioned groups. Answering these questions can improve the students’ academic status through training workshops. Thus, the current research was an attempt to determine the role of emotional intelligence in identification of self-efficacy among students with different levels of academic achievement.

## Methods

This descriptive correlation study was carried out on all the students of Kermanshah faculty of public health in 2011. The students were classified as low and high in academic achievement. According to the inclusion criteria of the study, the freshmen were not assessed. In the high and low groups, the average scores were >16 and <14, respectively. Data were collected through census method (except the freshmen). The sample consisted of 129 students, 98 females and 31 males. The students with high and low scores were 83 and 46, respectively. 

In this study, the measurement tool was standard Cyber-Shrink emotional intelligence questionnaire, and Persian Adaptation (Farsi) of the General Self-Efficacy Scale standardized by Nezami, Mitusi, Jar, Selm and Ralf (24). Cyber-Shrink is a self-evaluation questionnaire that measures 33 subjects and has five components of emotional intelligence, including self-awareness, self-control, spontaneity, social awareness and social skills. The questionnaire is designed based on Likert scale with scores ranging from 5 to 1 (5= always, 1= hardly). The scores of this questionnaire ranged from 33 to 165. General Self-Efficacy Scale of Nezami,  Schwarzer and Jerusalem is a ten-item questionnaire based on Likert scale with 5 choices from totally agree (score of 5) to completely disagree (score of 1). In the present study, Cronbach's alpha value was 87.7%. The questionnaires were sent to the students. To observe the ethical considerations, participation in the study was voluntary, and the questionnaires were filled out and collected anonymously. To perform the study based on the students’ record, the list of students with poor and good academic achievement was prepared; then, the required information was collected. For data analysis, SPSS 14 (SPSS Inc, Chicago, IL, USA) software was used and descriptive statistics, t-test, regression, and Analysis of Variance (ANOVA) were applied. P<0.05 was considered significant. 

## Results

A sample of 129 students (31 males and 98 females) aged 19-35 participated in the study. The mean age of the students was 22.03 ± 3.13 years; 33 (25.6%) of them, however, had not mentioned their age. 18.6% and 81.4% of the students were studying for associate degree and B.S., respectively. One person was excluded from the study due to incomplete questionnaire, and the data of 128 students were analyzed. 46 students gained a GPA of 14 and lower and 82 students acquired a GPA of 16 and higher.

The results of this study showed that the average score of students with high academic achievement was higher in emotional intelligence variables and their components than that of students with low academic achievement. In addition, the lowest and highest averages of both groups were associated with social skills and self-awareness components, respectively (Table 1).

**Table 1:** Mean and standard deviation of the scores of self-efficient beliefs and components of students’ emotional intelligence in high and low GPA levels

Grade	Components	Mean±SD
Low	Self-efficacy	39.17±5.91
	Self-motivation	23.02±3.33
	Self-awareness	28.54±3.57
	Self-control	21.78±3.68
	Social soberness	20.46±3.89
	Social skill	18.26±3.38
	Emotional intelligence	112.07±13.23
High	Self-efficacy	39.78±5.82
	Self-motivation	24.16±2.75
	Self-awareness	29.62±3.38
	Self-control	23.10±3.71
	Social soberness	21.79±2.87
	Social skill	19.11±3.18
	Emotional intelligence	117.7±10.33

The value of non-standardized squared correlation coefficient ​​showed that the total emotional intelligence score in students with low academic achievement explained 0.289 of self-efficacy, while the total emotional intelligence score in students with high academic achievement explained 0.409 of self-efficacy. Since the level obtained was significant (p<0.001), the applied model was significant, representing enough validity of the analysis (Table 2).

**Table 2:** Coefficients resulting from stepwise regression analysis of students’ scores in terms of emotional intelligence and self-efficacy

Grade	Model	Unstandardized coefficients		Standardized coefficients	R^2^	F	p
		B	SD. Error	Beta			
Low	(constant)	12.249	6.406	0.538	0.289	1.912	0.062
	Emotional intelligence	0.240	0.057			4.232	<0.001
High	(constant)	-2.701	5.782	0.640	0.409	-0.469	0.641
	Emotional intelligence	0.361	0.049			7.400	<0.001

The results of regression analysis regarding the role of emotional intelligence components in explaining self-efficacy indicated that 0.326 of self-efficacy structure was explained using self-awareness and self-motivation components in students with low academic achievement, and in students with high academic achievement, self-awareness, self-motivation and social soberness explained 0.458 of the variance associated with self-efficacy structure. Since the level obtained was significant (p<0.001), the applied model was significant with 90% confidence level, representing enough validity of the analysis (tables 2 and 3).

According to Table 3, based on Beta coefficient, each of the emotional intelligence components in regression equation contributed to the prediction of self-efficacy as follows:+(Constant) 8.549 = Self-efficacy of students with low academic achievement (Self-awareness components along with self-motivation) 1.179. The above equation shows that there is a relationship between the combination of the above-mentioned components and self-efficacy in students with low academic achievement, and self-efficacy of students with high academic achievement (self-awareness components along with students’ self-motivation and social consciousness) was 1.907. The above equation shows that there is a relationship between the combination of the above-mentioned components and self-efficacy in students with high academic achievement (Table 3).

**Table 3:** Coefficients resulting from stepwise regression analysis of students’ scores in terms of emotional intelligence and self-efficacy components

Grade	Model	Unstandardized coefficients		Standardized coefficients	t	p	R	R^2^
		B	SD .Error	Beta				
Low	(constant)	8.54	6.75	1.26	0.289	0.212	0.571	0.326
	Self-awareness	0.633	0.222	2.85	0.383	0.007		
	Self-motivation	0.633	0.238	2.29	0.308	0.027		
High	(constant)	-8.035	5.97	-1.34	0.409	0.183	0.677	0.458
	Self-awareness	0.536	0.171	3.14	0.311	0.002		
	Self-motivation	0.870	0.183	4.74	0.412	0.000		
	Social soberness	0.501	0.196	2.56	0.247	0.012		

## Discussion

The findings of the present study showed that the mean score of the students with high academic performance in emotional intelligence along with its components was higher than that of the students with poor academic performance. Moreover, the lowest mean score was reported for social skills and the highest mean score was reported for self-awareness component in both groups. In support of these findings, some studies have indicated that emotional intelligence is able to predict academic performance (9, 25). Due to this finding, it seems that students with high level of academic performance have acquired higher scores in emotional intelligence and its related components. The study carried out by Amini et al. showed that successful students had obtained higher scores than unsuccessful students in emotional intelligence variable (8). The studies not in line with the present study cast doubt on the predictive ability of emotional intelligence in academic performance and achievement (10, 26). This disagreement may be due to the presence of other variables involved in the academic achievement such as academic motivation, which has been shown to have a significant correlation with emotional intelligence reported in several studies (11).

The results showed that emotional intelligence in students with high academic achievement explained 40% of self-efficacy score. This was, however, around 29% for the students with low academic achievement. Studies have indicated that students with high emotional intelligence have higher academic achievement (8, 9, 25) and compatibility with different situations and possess the required skills to deal with the problems (12), which are considered as elements of self-efficacy (3). According to Bandura, self-efficacy is a socio-cognitive factor that can assist an individual in fulfilling the responsibilities (13). Self-efficacy is also predictive of academic achievement (15, 16). In addition, successful students achieved higher scores in components of emotional intelligence (27). Thus, it can be argued that students with high academic achievement obtain higher scores than those with low academic achievement in both variables of emotional intelligence and self-efficacy, as both variables are effective factors in academic achievement. That is why emotional intelligence is a better predictor of self-efficacy for the students with high academic achievement.

As to the potential of emotional intelligence components to predict self-efficacy and students’ academic achievement, the findings indicated that self-awareness and self-motivational components of emotional intelligence predicted 33% of the self-efficacy scores of the students with low academic achievement. On the other hand, the mentioned components and students’ self-efficacy had a significant correlation with low academic achievement. However, in students with high academic achievement, the self-awareness, self-motivational and socio-cognitive components explained approximately 46% of the variance associated with self-efficacy. In support of these findings, studies have shown a significant correlation between self-efficacy and final term scores (17) and academic achievement (15, 18). Since academic achievement and failure are associated with self-motivation and self-esteem (19), and emotional intelligence and self-efficacy have a high level of overlap (3) and a positively significant relationship with each other such that they can predict each other (20), it seems that emotional intelligence and its components are appropriate predictors of students’ self-efficacy in case students have academic motivation, self-esteem, self-efficacy and academic achievement, which are partly present in both groups of the students. However, they are better predictors in students with higher academic achievement. According to the findings obtained in this study, socio-cognitive component was a predictor of self-efficacy for the students with high academic achievement. This might be due to higher levels of social skills in students with higher achievement (28) and that socio-cognitive component is associated with social skills. Hence, self-efficacy and consequently academic achievement of the students can be promoted through educational interventions, aiming to improve the students’ emotional intelligence.

## Conclusions

The findings of the present study showed that emotional intelligence and self-efficacy play an important role in achieving academic success, and emotional intelligence can explain self-efficacy. Moreover, it is predicted that the feeling of self-efficacy in students will be increased by the rise in emotional intelligence. Therefore, it is recommended to teach emotional intelligence skills to students, especially those with low academic achievement in training workshops. 
